# Heterogeneity in Risk and Implications for Hepatitis C Reinfection in People Who Inject Drugs in England

**DOI:** 10.1111/jvh.14052

**Published:** 2025-01-16

**Authors:** Bryn Hilton, Daniela De Angelis, Holly Mitchell, Ross Harris

**Affiliations:** ^1^ MPhil Population Health Sciences, Department of Public Health and Primary Care University of Cambridge Cambridge UK; ^2^ MRC Biostatistics Unit, School of Clinical Medicine University of Cambridge Cambridge UK; ^3^ Blood Safety, Hepatitis, STI and HIV Division UK Health Security Agency London UK; ^4^ Statistics, Modelling and Economics Department UK Health Security Agency London UK

**Keywords:** hepatitis, hepatitis C, illicit drugs, reinfection, statistics

## Abstract

Chronic hepatitis C virus (HCV) infection is associated with significant morbidity, mortality and health economic burden. Over 90% of HCV cases in England occur in people who inject drugs (PWID). Current treatments for HCV are effective but do not protect against reinfection. This research characterised HCV infection and reinfection risk in PWID in England using 2011–2021 data from the annual, cross‐sectional, bio‐behavioural survey of PWID, Unlinked Anonymous Monitoring. Risk factors for HCV infection were explored using multivariable logistic regression. Shared frailty models for the force of infection (FOI) were used to estimate the risk of HCV infection throughout injecting career with unmeasured risk variation modelled using gamma‐shaped frailty distributions. HCV reinfection rates were derived using the frailty distributions of FOI models fitted to UAM data. Infection rates were highest in the first year of injecting (24 per 100 person‐years) but fell to between 5 and 8 infections per 100 person‐years subsequently. The estimated average annual risks of HCV primary infection and reinfection were 10.0% and 14.2%, indicating a 42% higher risk of reinfection compared to primary infection. Even those with no a priori risk factors were predicted to have high rates of reinfection if previously infected. These findings support the recognition of primary HCV infection as an independent risk factor for reinfection in PWID and emphasise the importance of reducing high‐risk behaviours to prevent HCV reinfection following treatment of primary infection. Public health policies must recognise the importance of preventing reinfection in efforts to reduce HCV infection prevalence.

## 1 Background

### 1.1 HCV Epidemiology

Hepatitis C virus (HCV) is a blood‐borne virus (BBV) characterised by inflammation of the liver [1]. Latest modelling estimates that in 2022, approximately 62,600 people were living with chronic HCV infection in England [2]. HCV is primarily spread through sharing of injecting equipment, as well as through sexual transmission. Most HCV infections occur in people who inject drugs (PWID). Of those living with chronic HCV, 20.1% of infections are estimated to have occurred in people who are currently injecting drugs, and 64.5% in those with a past history of injecting drugs, who are no longer injecting [2]. Since 2012, the estimated annual incidence for HCV infections in the United Kingdom has varied between 10 and 16 cases per 100 person‐years in PWID, without a clear directional trend [2].

The mainstay of current HCV treatment consists of direct‐acting antivirals (DAAs), a class of oral medications offering superior efficacy, improved tolerability and easier administration than the previously used interferon‐based treatment regimens [3]. Expanding access to DAAs has been identified as the primary reason for the reduction in HCV prevalence in England [2]. Although DAAs effectively cure HCV, they do not provide viral immunity and reinfection can occur if individuals continue high‐risk behaviours. In England, HCV reinfection rates have been estimated at 7.91 per 100 person‐years in the overall population and 10.5–22.5 per 100 person‐years in PWID [4, 5].

### 1.2 Frailty and Heterogeneity

The population of PWID is diverse, and the risk of contracting BBVs varies individual by individual. Some risk factors are readily measurable (e.g. sex, homelessness and needle‐sharing, etc.). Other ‘unmeasured factors’ have either not been measured (e.g. awareness of BBV risk) or are not readily measurable (e.g. injecting network, circumstances under which injecting behaviour began, genetic/immune susceptibility to infection, etc.). Unmeasured factors cannot be accounted for at the individual level when estimating HCV infection risk, yet still confer differential individual risk, often referred to as frailty. At the population level, frailty may be considered via a frailty distribution that represents the different infection risks of individuals attributed to unmeasured factors. The variance in a frailty distribution is termed heterogeneity [6, 7, 8].

Correlation between infections with a shared transmission route provides means to estimate heterogeneity within a population [6]. For PWID, frailty can be indirectly observed through correlation of BBVs (HCV, HBV and HIV). As BBVs share a common transmission route, individuals at higher risk of one BBV due to unmeasured factors will also be at higher risk of other BBVs [9, 10] The strength of within‐individual correlation between two BBVs offers a relative measure of population frailty for PWID. Individual‐level data can be used to estimate a risk distribution for the population, providing insight into the population's frailty distribution.

Population frailty distributions are typically represented through a statistical distribution, of which the gamma distribution is most used [7, 8, 11]. The gamma distribution is usually specified such that its mean equals 1, representing the average frailty in the population. Individuals with frailty > 1 have a higher than average infection risk, and conversely for individuals with frailty < 1. The variance in this gamma distribution represents a quantification of heterogeneity (Data S1).

**FIGURE 1:**
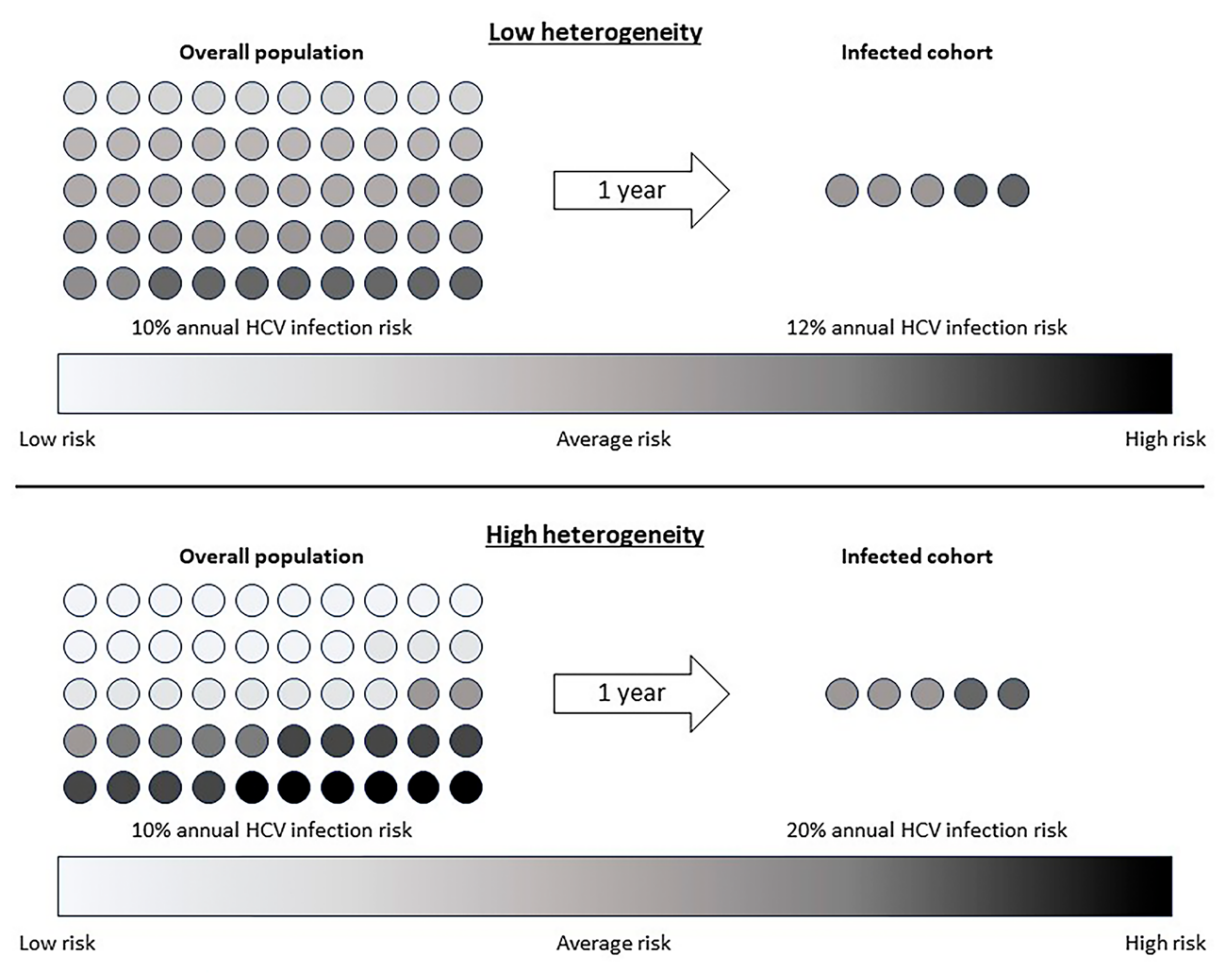
Average infection risk in two theoretical populations, with the same average population risk but different heterogeneity.

Quantifying heterogeneity gives insight into HCV reinfection risk. Consider two theoretical populations, both with the same average HCV infection risk but one with low heterogeneity and another with high heterogeneity (Figure 1). After 1 year, the same number of individuals in both populations will become infected. However, in the high heterogeneity population, the infected individuals are much more likely to have had high individual risk (high frailty). Conversely, the infected cohort from the low heterogeneity population has approximately the same average infection risk as the whole population. Assuming HCV treatment does not change risk behaviour, covariates that drive heterogeneity are associated with higher risk of reinfection. Understanding heterogeneity in PWID for primary HCV infection can shed light on theoretical HCV reinfection risk following treatment with DAAs.

Understanding HCV reinfection risk in PWID is critical to support ongoing HCV elimination efforts, in accordance with World Health Organization targets [12]. We aimed to offer novel insight into risk factors for HCV reinfection in PWID in England. First, we explored individual risk factors for HCV infection through multiple regression modelling. Second, we used force of infection (FOI) modelling to provide insight into population‐level frailty distributions for PWID. And finally, we estimated HCV reinfection rates through population‐level frailty distributions, conditional on prior primary infection and stable behavioural risk factors. We constructed models as time‐dependent to analyse the relationship between injecting career duration and reinfection risk.

## 2 Methods

### 2.1 Data Source

The Unlinked Anonymous Monitoring (UAM) survey is an annual, bio‐behavioural, cross‐sectional survey of PWID in England, Wales and Northern Ireland, co‐ordinated by the UK Health Security Agency (UKHSA), with support from Public Health Wales and the Public Health Agency for Northern Ireland [13]. Each year, a sample of specialist agencies working with PWID (drug treatment centres, needle exchange programmes and outreach work) recruit service users for UAM.

The survey has two distinct components: a dried blood spot (DBS) sample that is tested for HCV (RNA), HBV (surface antigen and antibodies to core antigen) and HIV (antibodies) (‘bio‐’) and a self‐completed questionnaire that covers demographics, social factors and questions related to injecting behaviour (‘behavioural’). Core questions have remained largely consistent over time, allowing longitudinal analysis of these factors [13].

This work included all survey responses corresponding to PWID in England between 2011 and 2021. Any records with missing data on viral status (HCV, HBV, or HIV) were excluded. All data are fully anonymised, with no patient‐identifiable information present. The dataset used in this research is summarised in Data S2.

### 2.2 Analysis Methods

Data analysis was performed in stages, with results of earlier stages informing covariate choice in later stages.

Odds ratios were calculated for all exposure variables and HCV status (measured using anti‐HCV, positivity representing ever having been infected with HCV). Significant (*p* < 0.05) variables were carried forward into multivariable logistic regression modelling to understand the relative importance of risk factors for HCV infection in PWID. The base model included all covariates with < 5% missing data. Sensitivity analyses were performed to assess the impact of broadening this eligibility criteria (Data S3). Multicollinearity was assessed by computing variance inflation factors for predictor variables [14]. Analysis of missing data is summarised in Data S4.

Force of infection (FOI) models were constructed based on UAM HCV‐HBV paired infection data and used to estimate the rate of HCV spread within a susceptible population of PWID. FOI is the instantaneous per capita rate at which susceptible individuals within a population become infected [8]. Within the framework of HCV infection in PWID, time is represented by an individual's ‘career duration’, the time in years since that person began injecting drugs. The FOI was assumed to change over time in accordance with the observation that individuals are at highest risk towards the beginning of their injecting career. HCV reinfection rates were derived using the frailty distributions of FOI models fitted to UAM data. This method relies on the assumption that individual frailty does not change with HCV treatment (i.e. the frailty distribution remains the same for primary infection and reinfection). The steps performed in FOI modelling and estimation of reinfection rates are summarised in Data S5.

Complete case analyses were performed using R, Version 4.3.0.

## 3 Results

A total of 24,269 (93.7%) records met eligibility criteria and were included in this analysis. Table 1 provides a descriptive summary and missing data proportions for eligible records.

**TABLE 1:** Descriptive summary and missing data proportions for primary analysis.

Variable	Available data	Missing data (%)
Viral status and treatment		
HCV status (anti‐HCV)	Positive: 11,673 (48.1%) Negative: 12,596 (51.9%)	0%
HBV status (anti‐HBc)	Positive: 3228 (13.3%) Negative: 21,041 (86.7%)	0%
Injecting behaviour		
Injecting duration	0–< 1: 1822 (9.0%) 1–< 3: 810 (4.0%) 3–< 5: 1316 (6.5%) 5–< 10: 3117 (15.4%) 10–< 15: 3785 (18.7%) 15+: 9392 (46.4%)	16.6%
Recent initiate	Yes: 2631 (13%) No: 17,609 (87%)	16.6%
Age at first use	< 18: 4048 (20.0%) 18–24: 7631 (37.7%) 25+: 8562 (42.3%)	16.6%
Injected drugs in the last month?	Yes: 8826 (41.9%) No: 12,239 (58.1%)	13.2%
Ever used needle exchange?	Yes: 21,641 (90.9%) No: 2167 (9.1%)	1.9%
Used needle exchange from the first year of injecting?	Yes: 9915 (50.5%) No: 9719 (49.5%)	19.1%
Days injecting per month	< 14 days injecting per month: 5248 (46.4%) 14+ days injecting per month: 6062 (53.6%)	53.4%
Use of crack cocaine (last 4 weeks)	Yes: 6186 (51.7%) No: 5779 (48.3%)	50.7%
Prescribed treatment for drug use?	Never prescribed: 2582 (10.8%) Previously prescribed: 3705 (15.5%) Currently prescribed: 17,618 (73.7%)	1.5%
Injected in the last year?	Yes: 6994 (30.4%) No: 16,013 (69.6%)	5.2%
Ever received needles or syringes from anyone? (2017–2021)	Yes: 5389 (62.2%) No: 3275 (37.8%)	64.3%
Sexual history		
MSM (1+ male partners in the last year)^a^	0 male partners: 16,248 (95.1%) 1+ male partners: 837 (4.9%)	29.6%
Number of sexual partners (last year)	0–1: 17,251 (74.2%) 2+: 5998 (25.8%)	4.2%
Condom use (last year)^b^	Never: 7149 (55.9%) Sometimes: 3709 (29.0%) Always: 1931 (15.1%)	47.3%
Received money, goods or drugs in exchange for sex (‘transactional sex’)	Never: 19,486 (86.8%) Yes, but not in the last year: 1706 (7.6%) Yes, in the last year: 1257 (5.6%)	7.5%
Environment		
Calendar time (year of survey)	2011: 2475 (10.2%) 2012: 2985 (12.3%) 2013: 2767 (11.4%) 2014: 2670 (11.0%) 2015: 2306 (9.5%) 2016: 2257 (9.3%) 2017: 2087 (8.6%) 2018: 2233 (9.2%) 2019: 2864 (11.8%) 2020: 510 (2.1%) 2021: 1141 (4.7%)	0%
Region	East of England: 1772 (7.3%) London: 3616 (14.9%) South East: 2937 (12.1%) South West: 2597 (10.7%) West Midlands: 2475 (10.2%) North West: 3543 (14.6%) Yorkshire and the Humber: 2645 (10.9%) East Midlands: 2767 (11.4%) North East: 1942 (8.0%)	0%
Sex	Male: 17,497 (72.9%) Female: 6505 (27.1%)	1.1%
Ever been to prison or a young offenders institute	Yes: 7292 (31.2%) No: 16,079 (68.8%)	3.7%
If you have been to prison or a young offenders institute, did injecting commence beforehand?	Yes: 7408 (52.9%) No: 6596 (47.1%)	42.3%
Homelessness	Yes, previous or current: 17,734 (75.8%) Never: 5662 (24.2%)	3.6%

*Note:* Missing data analyses are presented in Data S4.

^a^ Results are for male respondents only.

^b^ Results groups by number of partners in the last year were as follows: 0–1 partners: Never 11,851 (68.7%), Sometimes 3364 (19.5%), Always 2036 (11.8%); 2+ partners: Never 2231 (37.2%), Sometimes 2591 (43.2%), Always 1176 (19.6%).

### 3.1 Multivariable Logistic Regression

The base multivariable logistic regression model identified a number of ‘high‐risk’ characteristics that increased an individual's likelihood of acquiring HCV: female, homelessness (prior or current), having been to prison/young offenders institute, use of needle exchange and ever received drug treatment (Table 2). Sensitivity analyses also identified additional significant characteristics: injecting drugs in the last year, receiving money, goods or drugs in exchange for sex, sharing drug needles or syringes, calendar year and injecting duration. The risk of ever‐HCV infection increased with prolonged injecting duration, reflecting the cumulative risk of HCV infection PWID experience over time. MSM was not found to confer increased risk of HCV infection. More than a single sexual partner in the past year was associated with a reduced risk of HCV infection. The variance inflation factor across the base model and all sensitivity analyses was low (< 1.35 for all predictors), indicating that multicollinearity was not a problem in any of the regression models.

**TABLE 2:** Primary analysis results from univariate and multivariate logistic regressions in the base model.

Variable	Unadjusted odds ratio^a^ (95% confidence interval)	Adjusted odds ratio^b^ (95% confidence interval)
Calendar time (year of survey)		
2011	2011: 1.00 (reference)	2011: 1.00 (reference)
2012	1.17 (1.05, 1.30)	1.18 (1.05, 1.33)***
2013	1.21 (1.08, 1.35)	1.34 (1.19, 1.51)**
2014	1.22 (1.09, 1.37)	1.36 (1.21, 1.53)***
2015	1.28 (1.14, 1.44)	1.36 (1.20, 1.55)***
2016	1.37 (1.22, 1.55)	1.47 (1.30, 1.70)***
2017	1.40 (1.24, 1.59)	1.56 (1.37, 1.77)***
2018	1.51 (1.34, 1.71)	1.51 (1.33, 1.72)***
2019	1.47 (1.31, 1.65)	1.61 (1.42, 1.81)***
2020	1.74 (1.41, 2.15)	1.92 (1.54, 2.40)***
2021	1.58 (1.36, 1.84)	1.96 (1.67, 2.32)***
Region		
East of England	1.00 (reference)	1.00 (reference)
London	1.89 (1.68, 2.13)	2.07 (1.83, 2.35)***
South East	1.72 (1.52, 1.95)	1.74 (1.53, 1.98)***
South West	1.16 (1.02, 1.31)	1.25 (1.09, 1.43)**
West Midlands	0.81 (0.71, 0.92)	0.80 (0.70, 0.92)**
North West	2.32 (2.05, 2.62)	2.53 (2.23, 2.88)***
Yorkshire and Humber	1.47 (1.29, 1.66)	1.46 (1.27, 1.66)***
East Midlands	1.15 (1.02, 1.31)	1.08 (0.95, 1.23)
North East	0.79 (0.69, 0.91)	0.80 (0.70, 0.93)**
Sex		
Male	1.00 (reference)	1.00 (reference)
Female	0.96 (0.90, 1.02)	1.18 (1.11, 1.26)***
Homelessness		
Never	1.00 (reference)	1.00 (reference)
Yes, previous or current	1.82 (1.71, 1.94)	1.45 (1.36, 1.56)***
Ever been to prison or a young offenders institute?		
No	1.00 (reference)	1.00 (reference)
Yes	2.39 (2.25, 2.53)	2.15 (2.02, 2.30)***
Prescribed treatment for drug use?		
Never prescribed	1.00 (reference)	1.00 (reference)
Previously prescribed	2.31 (2.07, 2.58)	1.74 (1.55, 1.95)***
Currently prescribed	2.52 (2.30, 2.76)	1.75 (1.58, 1.93)***
Ever used needle exchange?		
No	1.00 (reference)	1.00 (reference)
Yes	3.84 (3.45, 4.28)	3.50 (3.13, 3.93)***
Number of sexual partners (last year)		
0–1	1.00 (reference)	1.00 (reference)
2+	0.79 (0.75, 0.84)	0.81 (0.76, 0.87)***

^a^ Odds ratio from univariate logistic regression.

^b^ Odds ratio from multivariate logistic regression, including all variables in the model.

*Note:* Significance notation for multivariable regression models: *p < 0.05; **p < 0.01; ***p < 0.001. Significance for univariate regression not shown.

### 3.2 Force of Infection

Estimates of the FOI consistently demonstrated that the risk of HCV infection was greatest in the first 6 months of injecting, followed by a gradually declining risk until 15 years of injecting duration (Table 3). Following this, in the 15+ injecting duration bands, there was a rise in FOI. Due to the nature of cross‐sectional survey data without a consistent cohort over time, infection prevalence can fall year‐on‐year due to chance, leading to falsely low FOI estimates during certain injecting duration bands.

**TABLE 3:** HCV injecting duration‐specific FOIs and population frailty variance for overall cohort and subgroups.

	Injecting duration‐specific force of infection						Frailty variance
	< 0.5 years	0.5–3 years	3–5 years	5–10 years	10–15 years	15+ years	
Overall	0.542	0.077	0.074	0.068	0.053	0.150	1.066
Sex							
Male	0.612	0.089	0.000^a^	0.098	0.084	0.104	0.766
Female	0.488	0.079	0.079	0.066	0.040	0.170	1.162
Homelessness							
Yes, previous or current	0.600	0.086	0.078	0.079	0.061	0.144	0.969
Never	0.403	0.066	0.037	0.042	0.004^a^	0.141	1.376
Ever been to prison or a young offenders institute							
Yes	0.711	0.099	0.095	0.054	0.067	0.142	0.925
No	0.365	0.075	0.000^a^	0.074	0.001^a^	0.123	1.446
Prescribed treatment for drug use?							
Never prescribed	0.195	0.074	0.105	0.034	0.054	0.133	1.494
Previously prescribed	0.555	0.055	0.125	0.067	0.037	0.133	0.971
Currently prescribed	0.706	0.085	0.023	0.078	0.048	0.156	1.051
Ever used needle exchange?							
Yes	0.748	0.049	0.065	0.071	0.050	0.151	1.028
No	0.251	0.050	0.106	0.028	0.021	0.088	2.252
Number of sexual partners (last year)							
0–1	0.637	0.073	0.089	0.061	0.050	0.156	1.066
2+	0.306	0.103	0.001^a^	0.090	0.061	0.127	1.045
Injected in the last year?							
Yes	0.504	0.082	0.081	0.086	0.022	0.135	0.940
No	0.578	0.074	0.043	0.032	0.147	0.203	1.480
Transactional sex							
Yes, in the last year	0.472	0.059	0.210	0.049	0.142	0.017	0.710
Yes, but not in the last year	0.290	0.111	0.122	0.081	0.086	0.079	0.817
Never	0.534	0.078	0.063	0.066	0.046	0.162	1.116
Ever received needles or syringes from anyone?							
Yes	0.702	0.111	0.151	0.130	0.003^a^	0.109	0.629
No	0.488	0.085	0.103	0.019	0.045	0.083	0.897

^a^ Anomalous result due to apparent reduction in HCV prevalence during this age band leading to low estimates of the FOI.

Results revealed that high‐risk characteristics (e.g. having ever been to prison) are associated with greater FOIs, regardless of injecting duration (i.e. an individual with a high‐risk characteristic is at increased risk of HCV infection throughout their injecting career). However, high‐risk characteristics also demonstrated lower frailty variances than their low‐risk counterparts.

The Akaike information criteria for the FOI models of the overall population that were frailty‐dependent and frailty‐independent were 19,857 and 20,188, respectively, indicating that incorporation of population frailty distribution improved the model fit considerably and is important in describing a population's HCV risk over time.

### 3.3 Derived Reinfection Rates

The calculated FOIs were used to estimate reinfection risk (methods described in Data S5). The overall cohort was considered against those individuals who have ever acquired HCV. The overall population represents the risk of primary HCV infection. The subset of people who have ever acquired HCV represents the population at risk of HCV reinfection. PWID who have ever acquired HCV have a higher average individual frailty than the overall population of all PWID (Z_avg_ = 1.43 vs. Z_avg_ = 1.00, respectively). Reinfection risk was greater than the risk of primary infection in the overall population, regardless of injecting duration (Table 4).

**TABLE 4:** FOI prediction of HCV reinfection rates for PWID in the overall population.

Population	Annual HCV infection risk (%) while within injecting duration band						
	0–1 years	1–3 years	3–5 years	5–10 years	10–15 years	15+ years	Average
Overall population (risk of primary infection)	24.0	7.2	6.6	6.2	5.1	13.0	10.0
Infected population (risk of reinfection)	38.3	12.0	11.1	9.7	7.6	17.0	14.2
Ratio of reinfection risk to primary infection risk	1.60	1.67	1.68	1.56	1.49	1.31	1.42

Those with the highest risk of primary infection acquire HCV early in their career. As career duration increases, those with moderate risk acquire primary infection, diluting the risk profile of those susceptible to reinfection. Thus, the population of those who have versus have not acquired primary infection becomes more similar, and the ratio of reinfection to primary infection risk reduces.

High‐risk characteristics conferred greater risk of primary infection and reinfection (Table 5). However, due to the higher heterogeneity in low‐risk cohorts, some individuals in these low‐risk populations are at high risk of infection (higher individual frailty). The rates of reinfection in low‐risk populations were greater than the rates of primary infection in their high‐risk counterpart populations.

**TABLE 5:** FOI prediction of HCV reinfection rates for PWID, considered by homelessness covariate and by use of needle exchange covariate.

Population	Annual HCV infection risk (%) while within injecting duration band						
	0–1 years	1–3 years	3–5 years	5–10 years	10–15 years	15+ years	Average
Homelessness							
Currently or previously homeless							
Overall population (primary infection)	26.0	8.0	7.4	7.5	5.8	12.8	10.5
Infected population (reinfection)	39.7	12.5	11.5	10.9	8.2	16.0	14.0
Ratio of reinfection rate to primary infection rate	1.5	1.6	1.6	1.5	1.4	1.3	1.5
Never experienced homelessness							
Overall population (primary infection)	18.3	5.7	3.2	4.1	0.4^a^	11.8	7.6
Infected population (reinfection)	30.8	10.8	6.4	7.6	0.7^a^	17.4	12.6
Ratio of reinfection rate to primary infection rate	1.7	1.9	2.0	1.9	1.8	1.5	1.7
Needle exchange							
Has used needle exchange							
Overall population (primary infection)	25.9	4.7	6.2	6.5	4.8	13.3	9.9
Infected population (reinfection)	42.0	8.0	9.9	10.0	7.2	17.1	14.1
Ratio of reinfection rate to primary infection rate	1.6	1.7	1.6	1.5	1.5	1.3	1.4
Never used needle exchange							
Overall population (primary infection)	12.0	4.2	8.1	2.7	1.9	7.5	6.9
Infected population (reinfection)	38.4	10.6	17.2	6.1	4.5	15.7	13.9
Ratio of reinfection rate to primary infection rate	3.2	2.5	2.1	2.3	2.4	2.1	2.0

^a^ Anomalous result due to apparent reduction in HCV prevalence during this age band leading to low modelled FOI.

## 4 Discussion

### 4.1 Principal Findings

Multivariable regression modelling identified high‐risk characteristics for HCV infection: female, homelessness (prior or current), having been to prison/young offenders institute, using needle exchange, receipt of drug treatment, injecting in the last year, transactional sex, sharing injecting equipment and longer injecting duration. The apparent increased risk in HCV infections associated with the use of needle exchange programmes or receipt of drug treatment may represent unmeasured covariates (e.g. level of drug dependence) or indicate residual confounding.

Infection rates were highest in the first year of injecting (24 per 100 person‐years) but fell to between 5 and 8 infections per 100 person‐years subsequently. Early career factors driving infection risk may include chaotic life circumstances leading up to the commencement of injecting, younger age and lack of awareness concerning BBVs and local harm reduction services. Plateauing of risk may reflect resolution of these early career factors. High‐risk characteristics increased infection risk throughout career duration, but this effect is most pronounced in early years, possibly due to correlation with unmeasured factors.

The estimated average annual risks of HCV primary infection and reinfection were 10.0% and 14.2%, indicating a 42% higher risk of reinfection compared to primary infection. FOI modelling revealed that high‐risk groups were more homogenous, with less individual variation in HCV infection risk compared to low‐risk groups. The greater heterogeneity in low‐risk groups indicates that a larger proportion of their HCV infection risk comes from unmeasured factors. As such, a small proportion of individuals within low‐risk cohorts experience very high HCV risk. Even those with no a priori risk factors were still predicted to have high rates of reinfection if previously infected.

### 4.2 Findings in Context

Risk factors consistently identified as significant for HCV reinfection include drug use in the 12 months prior to treatment, ongoing drug use during or after treatment, younger age and MSM [15, 16, 17, 18, 19]. Higher background prevalence of HCV is a significant risk factor for HCV reinfection for individuals engaging in high‐risk injection practices (i.e. sharing needles), but not for individuals who avoid these practices [20]. Studies with shorter follow‐up durations report higher rates of reinfection than those with longer follow‐up durations, suggesting that the risk of reinfection is greater shortly after HCV treatment and declines over time [15, 16].

Two recent studies report HCV reinfection rates and risk factors in England [4, 5]. Askar et al. (2022) reported a reinfection rate of 10.5 per 100 person‐years, with a median time to reinfection of 1.37 years (range 0.1–4.0) after treatment in those reinfected. Younger age was the only risk factor identified to carry an elevated risk of reinfection. Hibbert et al. (2023) used national surveillance data and record linkage to identify cases of HCV reinfection in people receiving DAA treatment in England [5]. The authors report a reinfection rate of 7.91 per 100 person‐years in the overall population, with significantly greater rates in high‐risk subgroups: current injecting drug users (22.5 per 100 person‐years) and those with a history of incarceration (20.42 per 100 person‐years). Younger age was again associated with increased reinfection risk; females carried a reduced reinfection risk. These two studies report higher reinfection rates than those in pooled analyses from systematic reviews. This is likely due to pooled analyses incorporating data from DAA clinical trials, with trial populations typically being lower risk than real‐world populations, especially now that treatment is being rolled out in community settings [21].

The reinfection rates presented here are similar to those in Askar et al. (2022) and Hibbert et al. (2023); during the plateau phase of risk identified in this research (injecting years 1 to 15), the HCV reinfection rate was 7.6–12.0 cases per 100 person‐years in the overall cohort. Therefore, the results of our derivation appear to have external validity within the context of PWID in England. Furthermore, these results offer novel insight into the impact of behavioural and sociodemographic factors on HCV reinfection risk.

### 4.3 Significance of Findings

The principal significance of this research is to support the recognition that HCV reinfection risk in people who have previously acquired HCV is greater than HCV primary infection risk in a population that has never been infected. The generalisability of our findings outside of England is dependent upon the similarity between the country's PWID population characteristics and those in the UAM cohort, its harm reduction services and its availability and use of DAAs. Reinfection risk is likely to differ from our results most notably in settings in which DAAs are restricted to only individuals who are no longer injecting drugs or in which harm reduction services are either substantially more or less robust than in England.

The relationship between frailty and reinfection risk illustrated here may also apply to other subgroups of people at increased risk of HCV infection through sexual practices, such as MSM and individuals who are homeless [22, 23]. Therefore, it may also be reasonable to consider prior HCV infection as an independent risk factor for reinfection regardless of injecting status, although this extended recommendation would need further research to support.

This update to HCV risk stratification could have implications for local harm reduction service commissioning, as well as community engagement strategies. Commissioning targeted harm reduction services, including adequate provision of sterile needles and syringes, for people who have received treatment for primary HCV infection and who continue to inject drugs would enhance the effectiveness of treatment as prevention. Furthermore, those receiving DAA treatment could be encouraged to participate in HCV awareness‐raising activities or even to act as local community leaders supporting harm reduction services and health advocacy [24]. Community peer programmes are already being utilised in England for peer‐to‐peer education and ‘buddying’ services to support PWID to engage with HCV testing and treatment [25].

Linkage to care is vital, regardless of where HCV testing or screening is conducted. As the evidence for the decentralisation of HCV testing and treatment emerges and care pathways expand out from specialist centres to better support vulnerable communities, it will be important to ensure that care pathways are highly integrated and coordinated, particularly when considering the elevated risk of HCV reinfection.

Finally, these findings emphasise the importance of testing those with ongoing risk regularly to identify reinfection and ensure early diagnosis, which is important for effective treatment and reduces the risk of onward transmission. Overall, these measures would support ongoing efforts in England and worldwide, to achieve and maintain HCV elimination.

### 4.4 Strengths and Limitations

The UAM survey is one of the largest bio‐behavioural cross‐sectional surveys of PWID in the world and represents a substantial and reliable source of information, used to estimate trends in BBV prevalence over time. The UAM survey is self‐reported and is therefore subject to reporting and recollection biases, although there is no incentive for participants to provide false information, given that the UAM survey is anonymised.

The UAM survey primarily recruits through drug treatment centres and needle‐exchange programmes. Individuals not engaging with harm reduction services may have different risks of HCV infection, and the UAM may tend to select individuals with differing characteristics from the wider population of PWID. However, unlike in the risk of primary HCV infection, the risk of reinfection is only relevant to individuals who have been diagnosed with and treated for primary HCV infection. These individuals would have received contact with health services and are likely to have been offered harm reduction services if they continue to inject drugs. Therefore, PWID who have been infected and treated are more likely to be comparable to those recruited in the UAM.

The UAM dataset is based on repeated cross‐sectional observations. Individuals may be represented more than once in the dataset, at different time points in their injecting career. Treating each observation as unique will underestimate standard errors and could skew results towards those with repeat participation. It is important to note, however, that as the UAM dataset does not represent longitudinal observation of panel data, the casual relationship between significant associations reported here is uncertain. This is particularly true for factors such as needle exchange attendance and drug treatment, where risks while on/off treatment cannot be explored at the individual level.

We used force of infection models to quantify the heterogeneity of HCV risk in PWID. A simple piecewise constant function was used for modelling, and we did not calculate confidence intervals for the estimated quantities. This could be improved on via parametric models and confidence intervals obtained through bootstrap approaches. Nevertheless, the precise FOI is only of indirect interest, with the key focus being on the quantification of heterogeneity and the proof of concept that heterogeneity in risk will lead to reinfection rates that are higher than primary infection rates.

The exploration of reinfection rates is founded on the assumption that individuals who undergo treatment for HCV infection do not change their risk behaviour. Data from clinical trials of DAAs have suggested some reduction in risk behaviours for PWID who are treated with DAAs [26, 27]. However, these clinical trial populations were subject to selection bias, excluding individuals engaging in high‐risk behaviours. Thus, results are likely not generalisable to the UAM population considered here. Regardless of the true impact of DAA treatment on risk behaviours, our findings are intended to illustrate the importance that risk behaviour is minimised following treatment of primary infection to reduce reinfection risk. This is particularly true now that treatment of high‐risk individuals through community pathways is being actively pursued [28].

## 5 Conclusions

This research identified factors that increase an individual's risk of becoming infected with HCV and evaluated the impact of these factors over the course of the individual's injecting career. Individual heterogeneity was quantified using frailty models and compared between subgroups. FOI modelling revealed that high‐risk groups were more homogenous, with less individual variation in HCV infection risk compared to low‐risk groups. More recent initiates to injecting were at greater risk of reinfection following treatment than those who had been injecting for many years. These findings support the recognition of primary HCV infection as an independent risk factor for reinfection in PWID and emphasise the importance of reducing high‐risk behaviours to prevent HCV reinfection following treatment of primary infection.

## Conflicts of Interest

The authors declare no conflicts of interest.

## Supporting information


Data S1.

## Data Availability

The data that support the findings of this study are available on reasonable request from the UKHSA. The data are not publicly available due to privacy or ethical restrictions.
