# Supplementary material for: Heterogeneity in Risk and Implications for Hepatitis C Reinfection in People Who Inject Drugs in England
Source: J Viral Hepat. 2025 Jan 16;32(2):e14052. doi: 10.1111/jvh.14052 (PMC11736538; doi:10.1111/jvh.14052)
Supplement: Supplementary file 1 — Data S1. [file JVH-32-0-s001.docx]

# SUPPLEMENTARY DATA

## SUPPLEMENTARY DATA 1 – GAMMA FRAILTY DISTRIBUTIONS

Supplementary Figure 1: Gamma frailty distributions for a population of 10,000 individuals with mean=1 and variance, δ


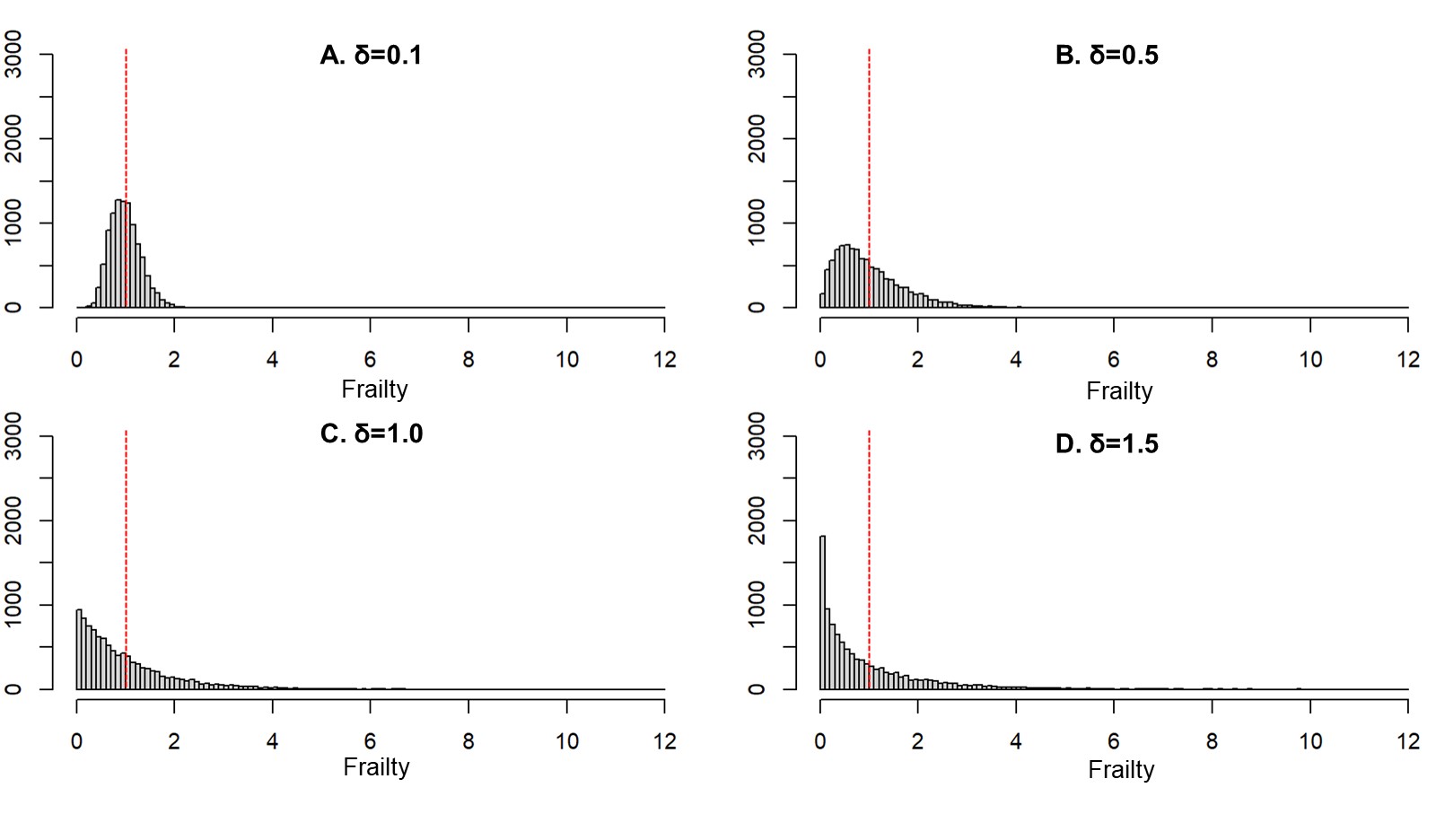


The red dashed lines indicate the average frailty (1) within the population.

## SUPPLEMENTARY DATA 2 – DATASET VARIABLES

Supplementary Table 1: Variables from the UAM survey included in the dataset

| Variable | Variable classification | Representation in UAM 2022 questionnaire |
| --- | --- | --- |
| Viral status and treatment | | |
| HCV status (anti-HCV) | Binary:   - Positive - Negative | N/A – DBS testing |
| HBV status (anti-HBc) | Binary:   - Positive - Negative | N/A – DBS testing |
| HIV status | Binary:   - Positive - Negative | N/A – DBS testing |
| HCV treatment | Binary:   - Yes - No | “Have you ever had treatment for your hepatitis C?” |
| Injecting history | | |
| Injecting duration (years) | Continuous data, categorised as below:   - 0–1 - 2 - 3–4 - 5–6 - 7–8 - 9–11 - 12–14 - 15–19 - 20–24 - 25–29 - 30–34 - 35+ | Calculated from the year of the UAM survey and the question: “How old were you when you first injected?” |
| Recent initiate | Binary:   - Yes - No | Calculated from injecting duration. Yes = <3-year injecting duration. No = 3+ year injecting duration. |
| Age at first use (years) | Continuous data, categorised as below:   - <18 - 18–24 - 25+ | “How old were you when you first injected [any drug]?” |
| Injected drugs in the last month | Binary:   - Yes - No | “Have you injected drugs in the last month?” |
| Sharing needles (ever) | Binary:   - Yes - No | “Have you ever injected with a needle or syringe that had already been used  by someone else (including your partner)?” |
| Sharing needles (last 28 days) | Binary:   - Yes - No | Calculated from:  “In the last month, to how many people have you passed on used needles or syringes (including your partner)?”  “In the last month, from how many people have you passed on used needles or syringes (including your partner)?” |
| Needle exchange (ever) | Binary:   - Yes - No | “Have you ever used a needle exchange (including a pharmacy exchange)?” |
| Days injecting per month | Binary   - <14 days injecting per month - 14+ days injecting per month | “In the last month, on how many days have you injected drugs?” |
| Use of crack cocaine (last year) | Binary:   - Yes - No | “In the last year, which of the following drugs have you injected?”  Crack cocaine is listed as an option. |
| Prescribed treatment for drug use? | Categorical:   - Never prescribed - Previously prescribed - Currently prescribed | “Have you ever been prescribed maintenance drug treatment (a script) (e.g., OST, methadone /buprenorphine)?” |
| Sexual history | | |
| MSM (last year) | Binary   - 0 male partners - 1+ male partners | “With how many men have you had sex with in the last year?” |
| Number of sexual partners (last year) | Binary:   - 0–1 - 2+ | Calculated from the following questions:  “With how many men have you had sex with in the last year?”  “With how many women have you had sex with in the last year?” |
| Condom use (last year) | Categorical:   - Always - Sometimes - Never | “How often did you use a condom when having sex in the last year?” |
| Transactional sex | Categorical:   - Never - Yes, but not in the last year - Yes, in the last year | “Have you ever received money, goods or drugs in exchange for sex?” |
| Environment | | |
| Calendar time (year of survey) | Categorical   - 2011 - 2012 - 2013 - 2014 - 2015 - 2016 - 2017 - 2018 - 2019 - 2020 - 2021 | Year of the UAM survey the observation was collected from |
| Region | Categorical:   - East of England - London - South East - South West - West Midlands - North West - Yorkshire and Humber - East Midlands - North East - Wales | Assumed to be the region in which the questionnaire was completed. |
| Sex | Binary:   - Male - Female | “Are you? [Male/Female]” ^†^ |
| Prison (ever) | Binary:   - Yes - No | “Have you ever been in prison (or a young offenders’ institution)?” |
| Prison (before injecting commencement) | Binary:   - Yes - No | Calculated from details of imprisonment (timing and duration), alongside age when injecting drugs commenced. |
| Homelessness | Categorical:   - Yes, not in the last year - Yes, in the last year - No | “Have you ever been homeless – that is living in a hostel, having no fixed above, or living on the streets?”  “Have you been homeless during the last year (12 months)?” |

Abbreviations: DBS: dried blood spot; MSM: men who have sex with men.

^†^Until 2019, this question was binary in the UAM survey. A third category ‘Other’ was introduced in the 2020 UAM survey. The ‘Other’ categorisation will not be explored given the low number of anticipated observations.

## SUPPLEMENTARY DATA 3 – MULTIVARIATE LOGISTICAL REGRESSION SENSITIVITY ANALYSES

**Multivariate logistic regression sensitivity analyses**

Supplementary Table 3: Multivariate logistic regression sensitivity analysis results

| Model | Exposure variable inclusion criteria | Exposure variables included | Number of records included  (% of all records) | AIC  (Base model AIC) | Notable differences from base model |
| --- | --- | --- | --- | --- | --- |
| Base model | <5% missing data | - Calendar time (year of survey) - Region - Sex - Homelessness - Ever been to prison or a young offenders institute? - Prescribed treatment for drug use? - Ever used needle exchange? - Number of sexual partners (last year) | 22,234 (91.6%) | N/A | N/A |
| Sensitivity analysis 1 | <10% missing data | - All exposure variables in base model - Injected in the last year? - Transactional sex | 20,558 (84.7%) | 25,854 (25,966) | Both additional included variables were significant:  Injected in the last year? – p<0.001  Transactional sex – p<0.01 |
| Sensitivity analysis 2 | <10% missing data + MSM | - All exposure variables in sensitivity analysis 1 - MSM | 14,954 (61.6%)^†^ | 18,884 (18,941) | MSM was not significant.  Inclusion of MSM did not alter the significance of transactional sex, suggesting that transactional sex is an HCV infection risk factor regardless of MSM. |
| Sensitivity analysis 3 | <10% missing data + Ever received needles or syringes from anyone?^1^ | - All exposure variables in sensitivity analysis 1 - Ever received needles or syringes from anyone? | 7,259 (29.9%)^‡^ | 9,022 (9,237) | The additional included variable was significant:  Ever received needles or syringes from anyone? – p<0.001 |
| Sensitivity analysis 4 | <10% missing data + (Year <2016) | - All exposure variables in sensitivity analysis 1 | 11,210 (46.2%)^§^ | N/A | No substantial differences from base model. |
| Sensitivity analysis 5 | <10% missing data + (Year ≥2016) | - All exposure variables in sensitivity analysis 1 | 9,348 (38.5%)^¶^ | N/A | Year was only significant in 2020 and 2021.  Transactional sex was no longer significant. |
| Sensitivity analysis 6 | <10% missing data + (Recent initiate = Yes)^2^ | - All exposure variables in sensitivity analysis 1 | 18,129 (74.7%) | 22,052 (22,966) | The additional included variable was significant:  Injecting duration – p<0.001 |

^†^Female PWID were excluded from this analysis as they are coded ‘NA’ in the UAM dataset. This sensitivity analysis therefore only included male PWID.

^‡^Data was only available for the variable “Ever received needles or syringes from anyone?” for the year 2017–2021. In this period, 82.1% of records were complete and used.

^§^For the period in this sensitivity analysis, 2011–2015, 85.0% of records were complete and used.

^¶^For the period in this sensitivity analysis, 2016–2021, 84.3% of records were complete and used.

Supplementary Table 4: Sensitivity analysis 6 – the effect of injecting duration on HCV infection

| Variable | Unadjusted odds ratio^†^  (95% confidence interval) | Adjusted odds ratio^‡^  (95% confidence interval) |
| --- | --- | --- |
| Injecting duration | - 0–<1 years: 1.00 (reference) - 1–<3 years: 1.68 (1.38, 2.04) - 3–<5 years: 2.07 (1.75, 2.45) - 5–<10 years: 2.59 (2.25, 2.98) - 10–<15 years: 4.09 (3.57, 4.70) - 15+ years: 7.15 (6.30, 8.13) | - 0–<1 years: 1.00 (reference) - 1–<3 years: 1.48 (1.20, 1.82)*** - 3–<5 years: 1.75 (1.46, 2.08)*** - 5–<10 years: 2.09 (1.81, 2.43)*** - 10–<15 years: 3.07 (2.66, 3.56)*** - 15+ years: 4.73 (4.12, 5.43)*** |

^†^Odds ratio from univariate logistic regression.

^‡^Odds ratio from multivariate logistic regression, including all variables in the model.

Significance notation for multivariable regression models: * = p<0.05; ** = p<0.01; *** = p<0.001. Significance for univariate regression not shown.

## SUPPLEMENTARY DATA 4 – MISSING DATA

Missing data proportions were highest in variables related to stigmatised sexual behaviour (MSM and condom use) and those related to risky injecting practices (crack cocaine use and high frequency injecting). Data for the question ‘Ever received needles or syringes from anyone?’ was only available for years 2017–2021, due to historical data integrity concerns prior to 2017. Multivariable logistic regression models of missing data showed numerous statistically significant associations between missing data for each variable, suggesting that data was not missing completely at random. The most notable pattern of missingness is within variables related to stigmatised sexual behaviour: number of sexual partners, condom use, MSM, and transactional sex.

Supplementary Figure 2: Multivariable regression analysis for missingness in variables

|  | | Outcome variable | | | | | | | | | | | | | | | | |
| --- | --- | --- | --- | --- | --- | --- | --- | --- | --- | --- | --- | --- | --- | --- | --- | --- | --- | --- |
|  |  | **Sex** | **InjDur** | **InjYear** | **Home** | **Share** | **Crack** | **InjMonth** | **Prison** | **Treat** | **Exchange** | **ExchYear** | **Day/Month** | **MSM** | **Partners** | **Condom** | **BeforePris** | **Transaction** |
| Exposure variables | **Sex** |  |  |  |  |  |  |  |  |  |  |  |  |  |  |  |  |  |
|  | **InjDur** |  |  |  |  |  |  |  |  |  |  |  |  |  |  |  |  |  |
|  | **InjYear** |  |  |  |  |  |  |  |  |  |  |  |  |  |  |  |  |  |
|  | **Home** |  |  |  |  |  |  |  |  |  |  |  |  |  |  |  |  |  |
|  | **Share** |  |  |  |  |  |  |  |  |  |  |  |  |  |  |  |  |  |
|  | **Crack** |  |  |  |  |  |  |  |  |  |  |  |  |  |  |  |  |  |
|  | **InjMonth** |  |  |  |  |  |  |  |  |  |  |  |  |  |  |  |  |  |
|  | **Prison** |  |  |  |  |  |  |  |  |  |  |  |  |  |  |  |  |  |
|  | **Treat** |  |  |  |  |  |  |  |  |  |  |  |  |  |  |  |  |  |
|  | **Exchange** |  |  |  |  |  |  |  |  |  |  |  |  |  |  |  |  |  |
|  | **ExchYear** |  |  |  |  |  |  |  |  |  |  |  |  |  |  |  |  |  |
|  | **Day/Month** |  |  |  |  |  |  |  |  |  |  |  |  |  |  |  |  |  |
|  | **MSM** |  |  |  |  |  |  |  |  |  |  |  |  |  |  |  |  |  |
|  | **Partners** |  |  |  |  |  |  |  |  |  |  |  |  |  |  |  |  |  |
|  | **Condom** |  |  |  |  |  |  |  |  |  |  |  |  |  |  |  |  |  |
|  | **BeforePris** |  |  |  |  |  |  |  |  |  |  |  |  |  |  |  |  |  |
|  | **Transaction** |  |  |  |  |  |  |  |  |  |  |  |  |  |  |  |  |  |

Multivariable logistic regression predicting the missingness of each outcome variable, using the presence (1) or absence (0) of observations in all other variables as exposure variables.

The key in Table 2 is presented below for interpretation of this figure.

Blue denotes positive effect (i.e., missingness in outcome is predicted by missingness in predictor; p<0.05).

Green denotes negative effect (i.e., missingness in outcome is predicted by completeness in predictor; p<0.05).

Supplementary Table 2: Variable key for Figure 2

| Abbreviated name | Full name |
| --- | --- |
| Sex | Sex |
| InjDur | Injecting duration |
| InjYear | Injected in the last year? |
| Home | Homelessness |
| Share | Ever received needles or syringes from anyone? |
| Crack | Use of crack cocaine (last 4 weeks) |
| InjMonth | Injected drugs in the last month? |
| Prison | Ever been to prison or a young offenders institute |
| Treat | Prescribed treatment for drug use? |
| Exchange | Ever used needle exchange? |
| ExchYear | Used needle exchange from the first year of injecting? |
| Day/Month | Days injecting per month |
| MSM | MSM (1+ male partners in the last year) |
| Partners | Number of sexual partners (last year) |
| Condom | Condom use (last year) |
| BeforePris | If you have been to prison or a young offenders institute, did injecting commence beforehand? |
| Transaction | Transactional sex |

## SUPPLEMENTARY DATA 5 - FOI MODELLING METHODOLOGY

The steps performed for FOI modelling were as follows:

- Injecting duration was categorised into 6 bands: 0–<0.5 years, 0.5–<3 years, 3–<5 years, 5–<10 years, 10–<15 years, and 15+ years.
- Each individual’s time spent in these bands was calculated based on their injecting duration.
- Hazards representing the rate at which each infection occurred during each time band were estimated, with parameters denoted as:
  - HCV: $h_{C}\left( t \right)$
  - HBV: $h_{B}\left( t \right)$
- To estimate time-specific FOIs, piecewise cumulative hazards, $A(t)$, were computed for each infection in each time band by integrating the respective hazards with respect to time. These integrals were computed as the sum of piecewise components. The piecewise constant FOI was divided into 6 time bands; for 2 viruses, this led to 12 model parameters.
  - HCV: $A_{C}\left( t \right)= \int_{0}^{t} h_{C}\left( x \right)dx$
  - HBV: $A_{B}\left( t \right)= \int_{0}^{t} h_{B}\left( x \right)dx$
- Frailty was modelled using a gamma distribution and was incorporated through an additional model parameter, θ, representing the shape of the gamma function; the rate parameter is also set to θ, such that the distribution has mean 1 and variance 1/θ. The probability of both HBV and HCV infections occurring ($\pi_{11}$), only HCV ($\pi_{10}$), only HBV ($\pi_{01}$), or neither infection occurring ($\pi_{00}$), were calculated from the cumulative hazards and θ. [1]
  - $\pi_{00}\left( t \right)=\left( 1+ \frac{A_{C}\left( t \right)+ A_{B}\left( t \right)}{\theta} \right)^{-\theta}$
  - $\pi_{10}\left( t \right)= \left( 1+ \frac{A_{B}\left( t \right)}{\theta} \right)^{-\theta}$-$\pi_{00}\left( t \right)$
  - $\pi_{01}\left( t \right)= \left( 1+ \frac{A_{C}\left( t \right)}{\theta} \right)^{-\theta}$-$\pi_{00}\left( t \right)$
  - $\pi_{11}\left( t \right)= 1- \pi_{C0}\left( t \right)- \pi_{0B}\left( t \right)- \pi_{00}\left( t \right)$
- Imperfect sensitivity of DBS HCV and HBV testing was accounted for, with a 98% sensitivity assumed for both infections based on data from two systematic reviews and metanalyses ($S_{1}=S_{2}=0.98$).[2]
  - $p_{00}\left( t \right)= \pi_{00}+(1-$ $S_{1}$) $\pi_{10}$ + (1$-$ $S_{2}$) $\pi_{01}+(1- S_{1})(1- S_{2})\pi_{11}$
  - $p_{10}\left( t \right)=$ $\pi_{10}S_{1}$ $+ S_{1}(1- S_{2})\pi_{11}$
  - $p_{0B}\left( t \right)= \pi_{01}S_{2}$ $+ S_{2}(1- S_{1})\pi_{11}$
  - $p_{CB}\left( t \right)= \pi_{11}S_{1}S_{2}$
- This 13-parameters were estimated through maximisation of the kernel log-likelihood for the data, which consisted of 2x2 multinomial data on HCV and HBV status at each injecting duration *t*, $n_{CBt}$, specified as:
  - $\sum_{t} \sum_{i,j=0}^{1} n_{CBt}log(p_{ij}(t))$

The Broyden–Fletcher–Goldfarb–Shanno algorithm was used for maximisation.

- The key outputs from each FOI model were:
  - 12 time-specific estimates of the FOIs (one for each injecting duration band, for each virus, λ). Only those pertaining to HCV infection are of direct relevance to this research.
  - An estimate of the variance δ of the frailty distribution, derived as 1/θ. This represents a quantification of the population *heterogeneity*.
- The probability of an individual with frailty Z being infected with HCV at time t, derived from

$$P_{HCV}(Z,t)=1-ⅇ^{-ZA_{C}\left( t \right)}$$

**Derivation of HCV reinfection rates**

HCV reinfection rates were derived numerically using the frailty distributions of FOI models fitted to UAM data. To do this, a new cohort of PWID was considered who are at risk of HCV reinfection. The individual frailties of members of this cohort were assigned based on random sampling of the UAM frailty distribution. Individual cumulative hazards were computed from injecting duration and the UAM population time-dependent FOI values. The probability of each member of this new cohort becoming reinfected with HCV could then be calculated. This method relies on the assumption that individual frailty does not change with HCV treatment (i.e., the frailty distribution remains the same for primary infection and reinfection). To appreciate the impact of heterogeneity on risk of reinfection, a high-risk subpopulation (PWID with a history of homelessness) was simulated and compared against its low-risk counterparts (PWID without a history of homelessness).

**References**

1. Farrington, C.P., et al., *Correlated infections: quantifying individual heterogeneity in the spread of infectious diseases.* Am J Epidemiol, 2013. **177**(5): p. 474-86.

2. Lange, B., et al., *Diagnostic accuracy of serological diagnosis of hepatitis C and B using dried blood spot samples (DBS): two systematic reviews and meta-analyses.* BMC Infect Dis, 2017. **17**(Suppl 1): p. 700.
